# MicroRNA mimics can distort physiological microRNA effects on immune checkpoints by triggering an antiviral interferon response

**DOI:** 10.1080/15476286.2022.2152978

**Published:** 2022-12-05

**Authors:** Felix Prinz, Katharina Jonas, Amar Balihodzic, Christiane Klec, Andreas Reicher, Dominik Andreas Barth, Jakob Riedl, Armin Gerger, Tobias Kiesslich, Christian Mayr, Beate Rinner, Julia Kargl, Martin Pichler

**Affiliations:** a Division of Oncology, Department of Internal Medicine, Medical University of Graz, Graz, Austria; b Research Unit for Non-Coding RNA and Genome Editing, Medical University of Graz, Graz, Austria; c Center for Physiology, Pathophysiology and Biophysics, Institute for Physiology and Pathophysiology Salzburg, Paracelsus Medical University, Salzburg, Austria; d Department of Internal Medicine I, University Hospital Salzburg, Paracelsus Medical University, Salzburg, Austria; e Division of Biomedical Research, Medical University of Graz, Graz, Austria; f Division of Pharmacology, Otto Loewi Research Center, Medical University of Graz, Graz, Austria; g Department of Experimental Therapeutics, the University of Texas MD Anderson Cancer Center, Houston, TX, USA

**Keywords:** MicroRNA mimics, miR-200c-3p, non-specific effects, dsRNA sensing, antiviral response, interferons, immune checkpoints

## Abstract

The microRNA-200 family has wide-ranging regulatory functions in cancer development and progression. Above all, it is strongly associated with the epithelial-to-mesenchymal transition (EMT), a process during which cells change their epithelial to a mesenchymal phenotype and acquire invasive characteristics. More recently, miR-200 family members have also been reported to impact the immune evasion of cancer cells by regulating the expression of immunoinhibitory immune checkpoints (ICs) like PD-L1. Therefore, we aimed to comprehensively characterize this miR-200 family as a regulatory interface between EMT and immune evasion mechanisms in biliary tract cancer. Initial correlation analyses and transient overexpression experiments using miRNA mimics suggested miR-200c-3p as a putative regulator of ICs including PD-L1, LGALS9, and IDO1. However, these effects could not be confirmed in stable miR-200c-3p overexpression cell lines, nor in cells transiently transfected with miR-200c-3p mimic from an independent manufacturer. By shifting our efforts towards dissecting the mechanisms leading to these disparate effects, we observed that the initially used miR-200c-3p mimic triggered a double-stranded (ds)RNA-dependent antiviral response. Besides upregulating the ICs, this had substantial cellular consequences including an induction of interferon type I and type III expression, increased levels of intracellular dsRNA sensors, and a significantly altered cellular growth and apoptotic activity.Our study highlights the capability of miRNA mimics to non-specifically induce a dsRNA-mediated antiviral interferon response. Consequently, phenotypic alterations crucially distort physiological miRNA functions and might result in a major misinterpretation of previous and future miRNA studies, especially in the context of IC regulation.

## Introduction

Since their discovery in 1993, microRNAs (miRNAs) have accumulated substantial recognition, owing to their role as fundamental regulators of health and disease [1,2]. They are small (18–25 nt), non-coding RNAs, which post-transcriptionally regulate the expression of a wide spectrum of genes in a sequence-dependent manner [3]. Since miRNAs significantly contribute to a balanced gene expression profile within a cell, it is clear that dysregulated miRNA levels frequently result in disease, including cancer [4,5].

There are numerous reports on how miRNAs impact cancer biology, either by acting as oncogenic or tumour-suppressive regulators [6]. They were reported to influence a plethora of cancer cell features, including cell proliferation, the establishment of chemoresistance, apoptosis regulation, and the epithelial-to-mesenchymal transition (EMT) [7–10].

EMT is a process during which cancer cells with an epithelial phenotype acquire mesenchymal characteristics, rendering them more migrative and invasive and ultimately promoting the formation of metastases [11]. This is highlighted by its important role at the invasive front of colorectal cancer and liver metastases, where EMT-related gene expression signatures including miRNAs were reported to predict patient survival [12,13]. Besides determining tumour cell motility, EMT has also been reported to impact the immune evasion of cancer cells by influencing the expression of immune checkpoints (ICs) [14–17]. Broadly speaking, ICs are proteins, which contribute to the establishment of an immunosuppressive microenvironment within tumours [18,19]. ICs include cell surface molecules on cancer cells (e.g. PD-L1, LGALS9), which interact with cognate receptors on effector immune cells (e.g. PD-1, TIM3), resulting in immune cell exhaustion [20,21]. Furthermore, also intracellular enzymes can act as immunosuppressive ICs. By hyperactively catabolizing molecules that are important for immune cell activity, enzymes like IDO1 lead to a metabolic inhibition of immune cells [22,23].

One miRNA family that is especially relevant in the context of EMT is the miR-200 family. This well-characterized miRNA family consists of the five members miR-200a, miR-200b, miR-429, miR-141, and miR-200c. Due to their negative regulation of the pro-mesenchymal transcription factor ZEB1, these miRNAs play a substantial role in controlling a cells’ EMT status [24,25]. Intriguingly, the miR-200 family has recently also been linked to immune evasion mechanisms by regulating the expression of PD-L1 in tumour cells [26].

Based on the previously mentioned reports, we hypothesized that EMT status, miR-200 family expression, and immune evasion of cancer cells might be connected through an intricate regulatory network. This seemed particularly interesting in the context of biliary tract cancer (BTC), since recent efforts in linking EMT and IC expression in gallbladder cancer have shown promising results [27]. Furthermore, latest results of a phase III clinical trial have underlined the value of IC therapy approaches in BTC, substantiating the need to identify molecular mechanisms behind IC regulation to allow further improvement of the still limited efficacy [28]. To this end, our initial aim was to investigate the effects of the EMT-regulating miR-200 family on the expression of ICs in BTC.

However, surprisingly, the focus of our study changed when we identified unintended miRNA mimic actions in the context of IC regulation. By non-specifically triggering an antiviral interferon response, miRNA mimics can induce unexpected wide-ranging phenotypic alterations, thereby critically distorting the interpretation of physiological miRNA functions.

## Materials and methods

### Cell culture

Human BTC cell lines HuCC-T1 (JCRB0425 [29]), HuH-28 (JCRB0426 [30]), OCUG-1 (JCRB0191 [31]), OZ (JCRB1032 [32]), NOZ (JCRB1033 [33]), KKU-055 (JCRB1551), KKU-100 (JCRB1568 [34]), KKU-213 (JCRB1557), and immortalized human cholangiocyte cell line MMNK-1 (JCRB1554 [35]) were purchased from the Japanese Collection of Research Bioresources Cell Bank (JCRB). Human BTC cell lines EGI-1 (ACC385) and TFK-1 (ACC344 [36]) were purchased from the German Collection of Microorganisms and Cell Cultures GmbH (DSMZ). All cell lines were cultured in L-glutamine-free Dulbecco’s Modified Eagle Medium (DMEM) containing 4.5 g/l glucose (Catalogue #: 11,960,044, Gibco, Thermo Fisher Scientific, Vienna, Austria). Medium was supplemented with 10% foetal bovine serum (FBS; Catalogue #: S-FBS-SA-015, Serana Europe GmbH, Pessin, Germany) and 1% Penicillin-Streptomycin (Catalogue #: P4333, Sigma-Aldrich Handels GmbH, Vienna, Austria). Cells were cultured under standard conditions (37°C, 21% O_2_, 5% CO_2_, and 98% humidity).

### Transient transfection

For transient miRNA overexpression, cells were seeded in 6-well plates and transfected with 10 nM Syn-hsa-miR-200c-3p miScript miRNA Mimic (Catalogue #.: MSY0000617, QIAGEN, Hilden, Germany) or 10 nM Syn-hsa-miR-141-3p miScript miRNA Mimic (Catalogue #: MSY0000432, QIAGEN) and 10 nM AllStars Negative Control siRNA (Catalogue #: 1,027,281; QIAGEN) as corresponding negative control. Additionally, cells were transfected with 10 nM mirVana miR-200c-3p mimic (Catalogue #: 4,464,066, Thermo Fisher Scientific) and 10 nM mirVana miRNA mimic Negative Control #1 (Catalogue #: 4,464,058, Thermo Fisher Scientific) as corresponding negative control. For transient ZEB1 knockdown, cells were seeded in 6-well plates and transiently transfected with 50 nM siRNA directed against ZEB1 (Hs_ZEB1_2 FlexiTube siRNA, Catalogue #: SI04272492, QIAGEN) or 50 nM AllStars Negative Control siRNA. All transfection procedures were carried out according to the fast-forward protocol of the HiPerFect Transfection Reagent (Catalogue #: 301,704, QIAGEN).

### Generation of stable miRNA overexpression cell lines

Cells were seeded into 12-well plates at a density of 100,000 cells per well. After 24 h of incubation under standard conditions, growth medium was replaced with medium containing 10 μg/ml Polybrene® (Catalogue #: sc-134,220, Santa Cruz Biotechnology Inc., Dallas, TX, USA) and 0.5% ViralEntry Transduction Enhancer (Catalogue #: G515, Applied Biological Materials, Vancouver, Canada). For viral transduction, 25 μl lentivirus harbouring a vector encoding genes for miR-200c-3p, a puromycin resistance enzyme, and GFP (LentimiRa-GFP-hsa-miR-200c-3p Virus, Catalogue #: mh15263, Applied Biological Materials) were added to the cells. Cells transduced with 25 μl lentivirus harbouring an empty vector encoding genes for puromycin resistance enzyme and GFP (Lenti-III-mir-GFP Control Virus, Catalogue #: m002, Applied Biological Materials) served as corresponding negative controls. Transduced cells were continuously selected with medium containing the cell line-dependent, sublethal concentration of Puromycin Dihydrochloride (Catalogue #: A1113803, Gibco, Thermo Fisher Scientific) for up to eight weeks. After that, transduced cells were sorted according to their GFP expression via flow cytometry. Cells with the highest GFP intensity (top 20% GFP signal) were cultured and used for subsequent assays.

### Reverse transcription quantitative PCR (RT-qPCR)

Total RNA was isolated from 70–90% confluent cells using Trizol™ Reagent (Catalogue #: 15,596,026, Thermo Fisher Scientific) according to the manufacturer’s instructions. Subsequently, 1 μg of total RNA was reversely transcribed with the miScript II RT Kit (Catalogue #: 218,161, QIAGEN) using the miScript HiFlex buffer to enable quantification of both miRNA and mRNA. RT-qPCR was performed on the LightCycler 480 (Roche, Mannheim, Germany) using the QuantiTect SYBR Green PCR Kit (Catalogue #: 204,145, QIAGEN). For the quantification of miRNA expression, Hs_miR-141_1 miScript Primer Assay (Catalogue #: MS00003507, QIAGEN), Hs_miR-200a_1 miScript Primer Assay (Catalogue #: MS00003738, QIAGEN), Hs_miR-200b_3 miScript Primer Assay (Catalogue #: MS00009016, QIAGEN), Hs_miR-200c_1 miScript Primer Assay (Catalogue #: MS00003752, QIAGEN) Hs_miR-429_1 miScript Primer Assay (Catalogue #: MS00004193, QIAGEN), and Hs_RNU6-2_11 miScript Primer Assay (Catalogue #: MS00033740, QIAGEN) were used, with RNU6-2 (=RNU6B) serving as reference gene. For mRNA quantification, the expression levels of E-cadherin, ZEB1, IDO1, LGALS9, and PD-L1 were normalized to the averaged expression of GAPDH and U6. Primer sequences are listed in Supplementary Table 1. Differences in gene expression were quantified by the ΔΔCt method.

### Immunoblot analysis

Cells were lysed in radioimmunoprecipitation assay (RIPA) buffer (150 mM NaCl, 50 mM Tris-HCl, 1% Triton X-100, 0.1% SDS, 0.1% sodium deoxycholate, 1% Nonidet P-40, pH 7.5) supplemented with 2% Protease Inhibitor Cocktail (Catalogue #: P8340, Sigma-Aldrich Handels GmbH, Vienna, Austria). For each sample, 30 µg of total protein were mixed with 4X Laemmli Sample Buffer (Catalogue #: 1,610,747, Bio-Rad Laboratories, Inc., Hercules, CA, USA) containing 10% β¯mercaptoethanol and denaturized at 95°C for 10 minutes. Proteins were separated on 4–15% Mini-PROTEAN® TGX Stain-free™ Gels (Catalogue #: 4,568,084, Bio-Rad Laboratories, Inc.) and transferred to a 0.45 µm nitrocellulose membrane (Catalogue #: 1,620,115, Bio-Rad Laboratories, Inc.). Membranes were blocked with 5% milk powder in Tris-buffered saline containing 0.1% Tween® 20 (TBS-T) for one to five hours before incubation with primary antibodies at 4°C for 16 hours. Membranes were washed three times with TBS-T and incubated with the corresponding secondary horseradish peroxidase (HRP)-conjugated antibodies for one hour at room temperature (RT). After three washing steps with TBS-T, membranes were incubated with SuperSignal™ West Pico PLUS Chemiluminescent Substrate (Catalogue #: 34,579, Thermo Fisher Scientific) or SuperSignal™ West Femto Maximum Sensitivity Substrate (Catalogue #: 34,094, Thermo Fisher Scientific). The chemiluminescent signals were imaged on a ChemiDoc™ Touch (Bio-Rad Laboratories, Inc.). To confirm equal protein loading, membranes were stripped with a stripping buffer containing 15 g/l glycine, 1 g/l sodium dodecyl sulphate (SDS), 1% Tween® 20, and pH 2.2 (according to the Abcam Mild stripping protocol) and re-probed with primary antibody directed against Cofilin at 4°C for 16 hours. After three washing steps, membranes were incubated with the corresponding secondary HRP-conjugated antibody for one hour at RT. Chemiluminescent signal was detected and imaged as described above. Used antibodies are listed in Supplementary Table 2.

### WST-1 assay

HuCC-T1 and OCUG-1 cells were seeded into 96-well plates at densities of 3,500 and 500 cells per well, respectively, and transfected with 10 nM of above-mentioned miR-200c-3p mimics and corresponding negative controls. 24, 48, 72, and 96 hours after transfection, 10 µL Cell Proliferation Reagent WST-1 (Cat. #: 11,644,807,001, Roche, Basel, Switzerland) was added to each well. After a 60-minute incubation at standard conditions, absorbances at 450 nm and 620 nm (reference wavelength) were measured on the SPECTROstar Omega (BMG LABTECH GmbH, Ortenberg, Germany).

### Caspase-Glo® 3/7 assay

HuCC-T1 and OCUG-1 cells were seeded into 96-well plates at densities of 3,500 and 500 cells per well, respectively, and transfected with 10 nM of above-mentioned miR-200c-3p mimics and corresponding negative controls. 72 hours after transfection, Caspase Glo® 3/7 Reagent (Cat. #: G8090, Promega, Madison, WI, USA) was added to the wells and the assay was performed according to the manufacturer’s instructions. One hour after reagent addition, luminescence was measured on LUMIstar Omega (BMG LABTECH GmbH, Ortenberg, Germany).

### Statistical analysis

All experiments were performed at least three independent times. Data is presented as mean + standard deviation (SD), unless otherwise indicated. Statistical analyses were performed using the GraphPad Prism 5.0 software (GraphPad Software, San Diego, CA, USA). Mann-Whitney U test, unpaired two-tailed t-test, and one-way ANOVA with Tukey’s Post Hoc test were used when applicable. P values below 0.05 were regarded as statistically significant. * p < 0.05, ** p < 0.01, *** p < 0.001.

## Results

### Endogenous expression of miR-200 family members correlates with the expression of EMT markers and immune checkpoints

**Figure 1.**
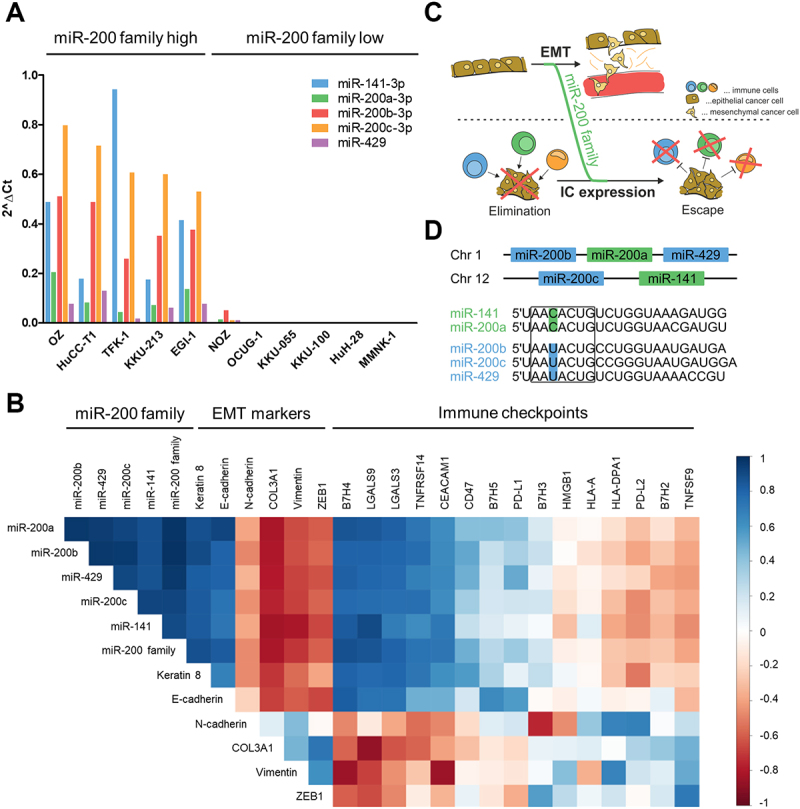
Endogenous expression of miR-200 family members correlates with the expression of EMT markers and IC molecules. (A) Endogenous miR-200 family member expression levels were analysed in ten BTC cell lines and the immortalized cholangiocyte cell line MMNK-1 with RT-qPCR to identify ‘miR-200 family high’ or ‘miR-200 family low’ cell lines. For mir-200c-3p, endogenous expression levels have been reported previously [37]. (B) Spearman Rank Correlation matrix depicting the correlations of miR-200 family member expression, the expression of EMT markers, and IC molecule expression across all eleven analysed cell lines. Expression data obtained by RT-qPCR. (C) Schematic outline of the initial hypothesis. (D) Diagram of the miR-200 family clusters. Seed sequences are framed. Different functional clusters are coloured green and blue.

As an initial step to explore a potential link between the EMT-regulating miR-200 family members and ICs, we analysed gene expression levels in ten BTC cell lines and the immortalized cholangiocyte cell line MMNK-1. The endogenous expression levels of miR-200 family members varied (Fig. 1A). Five of the eleven analysed cell lines displayed relatively high expression levels (categorized as miR-200 family high), while others had no detectable baseline expression (categorized as miR-200 family low). As previously reported for miR-200c-3p and EMT markers [37], we correlated the expression of miR-200 family members with the expression of certain EMT markers and ICs (Fig. 1B). The expression of miR-200 family members was positively correlated to epithelial markers E-cadherin and Keratin 8, while being negatively correlated to the mesenchymal markers N-cadherin, Collagen Type III Alpha 1 Chain (COL3A1), Vimentin, and Zinc finger E-box-binding homeoprotein 1 (ZEB1). Interestingly, miR-200 family member expression was positively correlated to the expression of several ICs, including V-Set Domain Containing T Cell Activation Inhibitor 1 (B7H4), Galectin 9 (LGALS9), Galectin 3 (LGALS3), Tumour Necrosis Factor Receptor Superfamily Member 14 (TNFRSF14), and Carcinoembryonic Antigen-Related Cell Adhesion Molecule 1 (CEACAM1). ZEB1 and other mesenchymal markers, however, were negatively correlated to those ICs. Therefore, we speculated that miR-200 family members not only influence EMT but also impact IC expression, thus serving as a potential link between these two cancer cell features (Fig. 1C).

The miR-200 family members can be divided into subfamilies either according to their genomic location (miR-200b/miR-200a/miR-429 located on chromosome 1, miR-200c/miR-141 located on chromosome 12) or based on their functionality as determined by the single-base alteration within the respective seed sequences (miR-141/miR-200a and miR-200b/miR-200c/miR-429) (Fig. 1D). Since miR-200c and miR-141 not only display high expression levels among the ‘miR-200 family high’ cell lines but also belong to different functional subfamilies, they were selected as representatives to study the connection between miR-200 family members, EMT, and the expression of ICs in BTC cells.

### Transfection of a specific miR-200c-3p mimic leads to an upregulation of the immune checkpoints LGALS9, PD-L1, and IDO1

**Figure 2.**
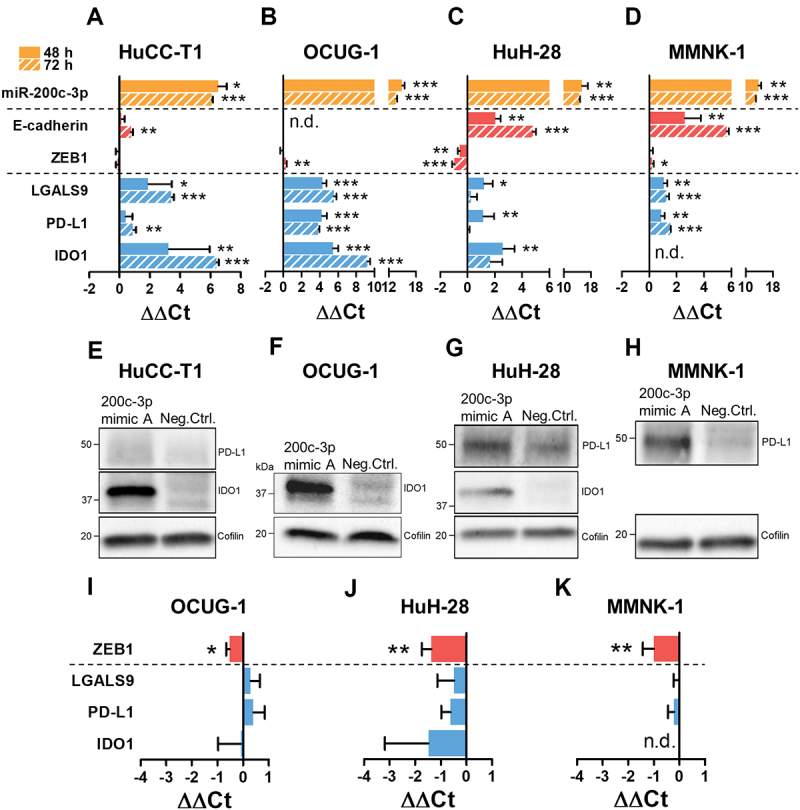
Transient miR-200c-3p mimic A transfection leads to the upregulation of LGALS9, PD-L1, and IDO1 expression in a ZEB1-independent manner. (A-D) HuCC-T1, OCUG-1, HuH-28, and MMNK-1 cells were transiently transfected with 10 nM miR-200c-3p mimic A (miScript) or AllStars Neg. Ctrl. for 48 hours (solid bars) or 72 hours (striped bars) and expression levels of miR-200c-3p, E-cadherin, ZEB1, LGALS9, PD-L1, and IDO1 were analysed via RT-qPCR. (I-K) OCUG-1 (n = 3), HuH-28 (n = 5), and MMNK-1 (n = 5) cells were transiently transfected with 50 nM ZEB1 siRNA or AllStars Neg. Ctrl. for 48 hours and expression levels of ZEB1, LGALS9, PD-L1, and IDO1 were analysed via RT-qPCR. RNU6B was used for normalization of miRNA levels, the mean of GAPDH+U6 for the normalization of mRNA levels. Differences in expression were evaluated using the ΔΔCt method. Data is presented as mean + SD. For **(a, c, d)** 48 h (n = 5) statistical analysis was performed using Mann-Whitney U test and for 72 h (n = 3) unpaired two-tailed t-test was used. For (B) 48 h (n = 3) and 72 h (n = 3), statistical analysis was performed using unpaired two-tailed t-test. For (I), statistical analysis was performed using unpaired two-tailed t-test, for (J, K) statistical analysis was performed using Mann-Whitney U test. *p < 0.05, **p < 0.01, ***p < 0.001. n.d. = not detectable (Ct≥35). **(e, f, h)** HuCC-T1, OCUG-1, and MMNK-1 cells were transfected with miR-200c-3p mimic A for 72 hours, (G) HuH-28 for 48 hours, and resulting changes in PD-L1 and IDO1 protein were analysed via Western Blot where applicable. Cofilin was used as loading control.

In order to study the relationship between miR-200 family members and ICs, we transiently overexpressed miR-141-3p or miR-200c-3p using miRNA mimics (miScript, QIAGEN; hereafter referred to as miRNA mimics A). Resulting changes in the expression levels of respective miRNAs, certain EMT markers, and a gene panel consisting of 21 ICs were analysed by RT-qPCR. While the overexpression of miR-141-3p did not lead to transcriptional alterations (Suppl. Fig. 1), miR-200c-3p overexpression resulted in a significant upregulation of LGALS9, Programmed Cell Death 1 Ligand 1 (PD-L1), and Indoleamine 2,3-Dioxygenase 1 (IDO1) on RNA level in all four analysed cell lines (Fig. 2A-D). These effects were confirmed on protein level. Along with the expected decreased protein levels of ZEB1 (Suppl. Fig. 2), cells transfected with miR-200c-3p mimic A displayed increased PD-L1 and IDO1 protein levels compared to cells transfected with the respective negative control (Fig. 2E-H).

In general, miR-200c-3p exerts its anti-EMT function by direct interaction and subsequent downregulation of the pro-mesenchymal transcription factor ZEB1. Therefore, we questioned whether the observed miR-200c-3p effect on the respective ICs was a consequence of a miR-200c-3p-induced ZEB1 downregulation or resulted from an independent mechanism. To this end, we silenced ZEB1 in OCUG-1, HuH-28, and MMNK-1 cells using siRNAs and analysed the impact on IC expression. Although we could confirm a sufficient siRNA-mediated downregulation of ZEB1, we did not observe relevant changes in the expression of LGALS9, PD-L1, or IDO1 (FIg. 2I-K). We concluded that miR-200c-3p overexpression leads to an upregulation of LGALS9, PD-L1, and IDO1 irrespective of its known interaction with ZEB1, and is driven by independent mechanisms.

### miR-200c-3p mimic A influences expression of PD-L1, LGALS9, and IDO1 by non-specific effects

**Figure 3.**
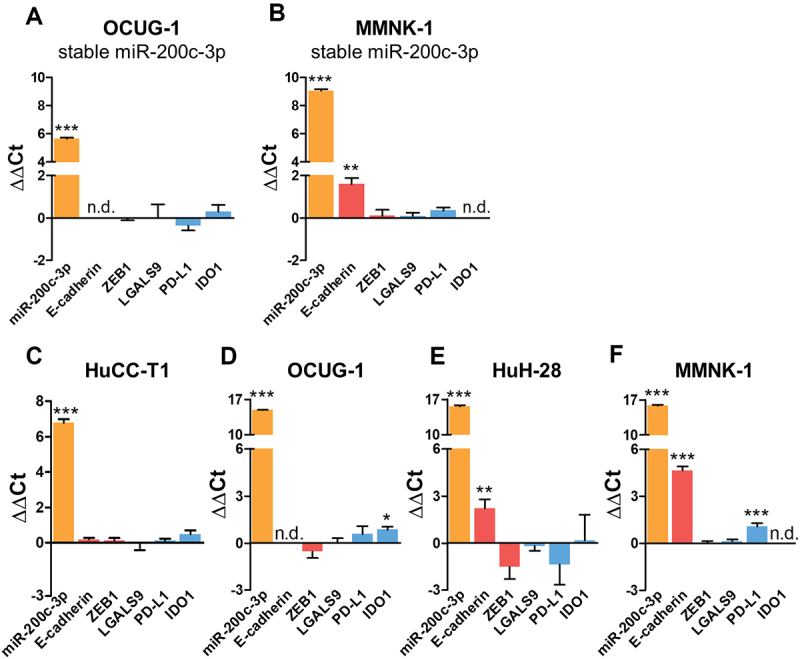
There is no upregulation of LGALS9, PD-L1, or IDO1 in stable miR-200c-3p overexpression cells nor in cells with transient miR-200c-3p overexpression using mirVana miR-200c-3p mimic B. (A, B) OCUG-1 (n = 3) and MMNK-1 (n = 3) cells were transduced with a miR-200c-3p overexpression lentivirus or a control lentivirus. (C-F) HuCC-T1 (n = 3), OCUG-1 (n = 3), HuH-28 (n = 3), and MMNK-1 (n = 3) cells were transiently transfected with 10 nM mirVana miR-200c-3p mimic B or mimic Neg. Ctrl. B for 48 hours. Expression levels of miR-200c-3p, E-cadherin, ZEB1, LGALS9, PD-L1, and IDO1 were analysed via RT-qPCR. RNU6B was used for normalization of miRNA levels, the mean of GAPDH+U6 for the normalization of mRNA levels. Differences in expression were evaluated using the ΔΔCt method. For MMNK-1 cells, data of miR-200c-3p, E-cadherin, and ZEB1 expression has been reported previously [37]. Data is presented as mean + SD. Statistical analysis was performed using unpaired two-tailed t-test. *p < 0.05, **p < 0.01, ***p < 0.001. n.d. = not detectable (Ct≥35).

In order to confirm the effects of transient miR-200c-3p overexpression on ICs in an additional experimental model system, we generated stable miR-200c-3p overexpression BTC cell lines by lentiviral transduction. Surprisingly, and in stark contrast to the results from the transient miR-200c-3p overexpression, we did not observe an upregulation of LGALS9, PD-L1, or IDO1 in the stable miR-200c-3p overexpression cells (Fig. 3A, B).

Given these unexpected disparities, we hypothesized that the IC upregulation upon transient miR-200c-3p mimic A transfection was not a physiologic effect conferred by the mature miR-200c-3p, but rather a non-specific effect triggered by the miR-200c-3p mimic A molecule. To address this concern, we transfected cells with a miR-200c-3p mimic from a different company (mirVana, Thermo Fisher Scientific; hereafter referred to as miR-200c-3p mimic B). While both mimics contain the same mature miR-200c-3p sequence, there are company-dependent differences in the composition of the mimic molecules. According to the manufacturers, miR-200c-3p mimic A is an unmodified double-stranded RNA oligonucleotide, whereas miR-200c-3p mimic B additionally contains chemical modifications to limit non-specific effects. By comparing transcriptional changes induced by miR-200c-3p mimic A and mimic B, this allowed us to determine whether the mature miR-200c-3p or only the particular miR-200c-3p mimic A molecule was responsible for the effects on respective ICs. Strikingly, although similar miR-200c-3p overexpression levels as with the miR-200c-3p mimic A were reached, miR-200c-3p mimic B transfection did not result in comparable effects on LGALS9, PD-L1, or IDO1 (Fig. 3C-F). This supports our theory that the miR-200c-3p mimic A molecule triggers the upregulation of ICs by a non-specific effect, independent of the physiologic miR-200c-3p function.

### miR-200c-3p mimic A triggers a dsRNA-dependent antiviral response program and leads to the upregulation of type I and type III interferons

**Figure 4.**
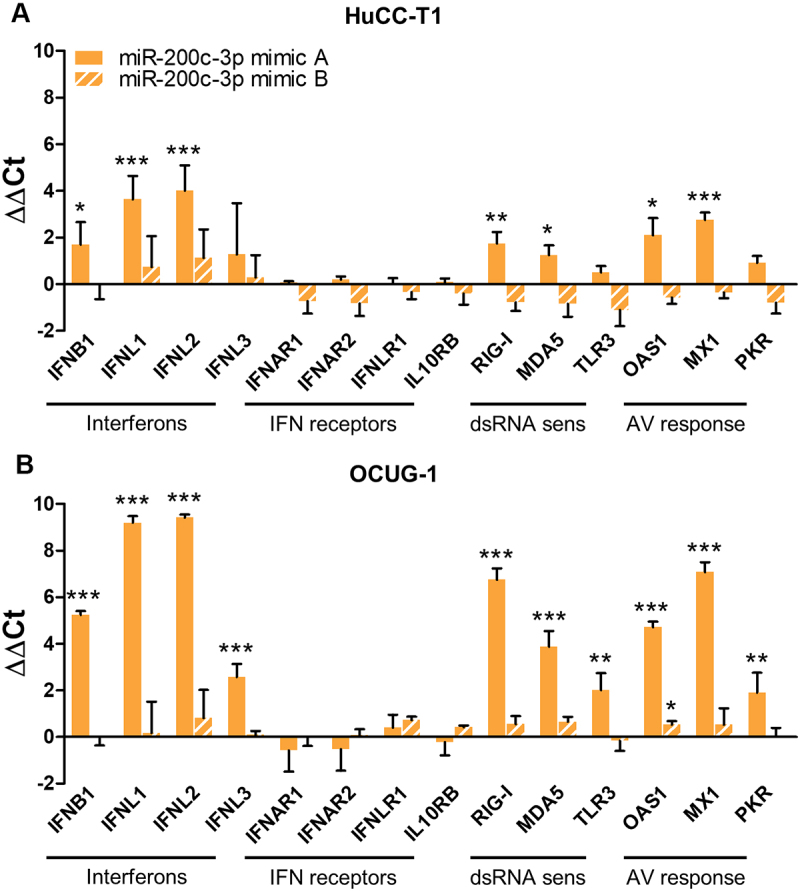
miScript miR-200c-3p mimic A, but not mirVana miR-200c-3p mimic B, triggers the upregulation of interferons, dsRNA sensors, and antiviral response genes. (A) HuCC-T1 (n = 3) and (B) OCUG-1 (n = 3) cells were transiently transfected with 10 nM miScript miR-200c-3p mimic A (solid bars) or mirVana miR-200c-3p mimic B (striped bars) and corresponding negative controls for 48 hours. Expression levels of interferons IFNB1, IFNL1, IFNL2, IFNL3, interferon receptors IFNAR1, IFNAR2, IFNLR1, IL10RB, double-stranded RNA sensors RIG-I, MDA5, TLR3, and antiviral response genes OAS1, MX1, PKR were analysed via RT-qPCR. The mean of GAPDH+U6 was used for the normalization of mRNA levels. Differences in expression were evaluated using the ΔΔCt method. Data is presented as mean + SD. Statistical analysis was performed using unpaired two-tailed t-test. *p < 0.05, **p < 0.01, ***p < 0.001. IFN: interferon, dsRNA sens: double-stranded RNA sensors, AV: antiviral.

In a first attempt to explain the contrasting results between miR-200c-3p mimic A and mimic B transfections, we looked at innate immune responses. We speculated that the uptake of the exogenous double-stranded miRNA mimic A triggers the cells’ antiviral programme, thereby causing the observed effects on ICs. To follow up on this hypothesis, we applied a RT-qPCR screening panel including genes involved in interferon (IFN) signalling, double-stranded RNA (dsRNA) recognition, and antiviral response. The transfection with the miR-200c-3p mimic A, but not with miR-200c-3p mimic B, resulted in a strong upregulation of the type I interferon IFNB1, the type III interferons IFNL1, IFNL2, and IFNL3, the dsRNA sensors DExD/H-Box Helicase 58 (RIG-I), Interferon Induced With Helicase C Domain 1 (MDA5), and Toll-Like Receptor 3 (TLR3), and the antiviral response genes 2’-5’-Oligoadenylate Synthetase 1 (OAS1), MX Dynamin Like GTPase 1 (MX1), and Eukaryotic Translation Initiation Factor 2 Alpha Kinase 2 (PKR) in HuCC-T1 and OCUG-1 cells. The expression levels of IFN receptors were not influenced (Fig. 4).

Of note, we did not observe a notable upregulation of interferons in cells transfected with miScript miR-141-3p mimic A (Suppl. Fig. 3a, b). This demonstrates that the non-specific effects are not triggered by any random miScript miRNA mimic, but seem to be dependent on additional factors. Furthermore, there was no upregulation of interferons in the stable miR-200c-3p overexpression cell lines (Suppl. Fig. 3c, d), corroborating the assumption that cells upregulate their antiviral defences specifically as a reaction to the exogenous miR-200c-3p mimic A molecule. Importantly, the concentration of the transfected miR-200c-3p mimic A mattered in the context of IC upregulation and interferon response. While there was no effect on neither ICs nor IFNs with concentrations up to 2.5 nM, higher concentrations led to an upregulation in a concentration-dependent manner (Suppl. Fig. 4).

### miR-200c-3p mimic A influences cell growth and apoptosis in an miR-200c-3p-unrelated manner

**Figure 5.**
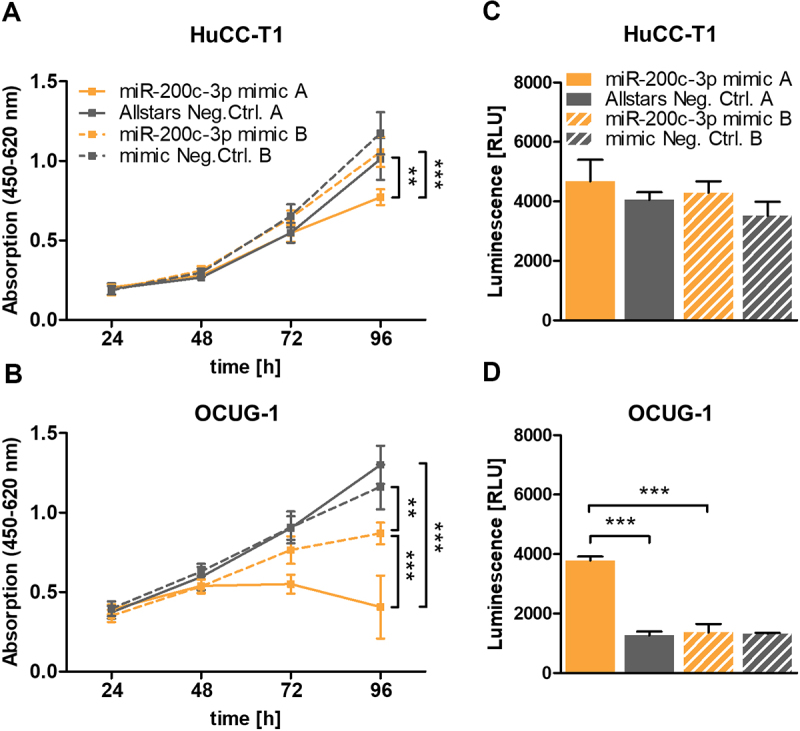
miScript miR-200c-3p mimic A and mirVana miR-200c-3p mimic B have different effects on cell growth and apoptosis. HuCC-T1 and OCUG-1 cells were transiently transfected with 10 nM miScript miR-200c-3p mimic or mirVana miR-200c-3p mimic, and corresponding negative controls and (A, B) proliferation was analysed every 24 hours over a duration of 96 hours using a WST-1 assay (n = 6) or (C, D) induction of apoptosis was analysed after 72 hours by a Caspase 3/7 Glo assay (n = 4). Data is presented as mean +SD. Statistical analysis was performed using one-way ANOVA with Tukey post-test. **p < 0.01, ***p < 0.001. RLU: Relative light unit.

Previous reports have shown that antiviral response is accompanied by reduced cell growth owing to the frequent induction of apoptosis [38]. Therefore, we analysed the effect of the different miR-200c-3p mimics on cellular growth to determine if miR-200c-3p mimic A influences further cellular aspects apart from the IC upregulation. Although miR-200c-3p mimic B transfection led to slightly reduced cell numbers after 96 hours, there was a significantly stronger inhibitory effect with miR-200c-3p mimic A. This indicates that although a potential physiological miR-200c-3p effect was observable, it was overpowered by an interferon-mediated inhibition of cell growth (Fig. 5A, B). In line with these results, we noticed a strong apoptosis induction in miR-200c-3p mimic A-transfected OCUG-1 cells (Fig. 5D). Interestingly, this effect was not visible in HuCC-T1 cells transfected with miR-200c-3p mimic A, indicating a potential perturbation of the cell cycle progression as an additional mechanism affecting cell growth (Fig. 5C).

## Discussion

The initial aim of the study was to comprehensively characterize the miR-200 family as a potentially pivotal coordinator between EMT and immune evasion in BTC. This seemed intriguing, because [1] miR-200 family expression and EMT status are tightly connected [24,25]; [2] members of the miR-200 family have already been mentioned to influence the expression of several ICs [26]; [3] it has been reported that EMT status of cancer cells predicts the response of patients to IC blockade [16]; and [4] miR-200c-3p expression was identified as prognostic biomarker for colorectal cancer, suggesting a key role for this miRNA in regulating the EMT-immune crosstalk [39].

After preliminary correlation analyses, which suggested a positive association of miR-200 family member expression and the endogenous levels of ICs, we set out to further explore this connection. When we transiently overexpressed miR-200c-3p using miScript miR-200c-3p mimic A, we observed a robust upregulation of the three ICs PD-L1, LGALS9, and IDO1 across all four cell lines tested. However, this initial finding could not be confirmed in stable miR-200c-3p overexpression cell lines nor when using a miR-200c-3p mimic B from a different manufacturer. Both of these overexpression settings failed to induce PD-L1, LGALS9, and IDO1 expression and made us question the validity of our initial findings. Reports of contrasting results further fuelled these doubts. Independent studies have stated that miR-200c-3p overexpression resulted in a downregulation of PD-L1 and IDO1 in adipose-derived mesenchymal stem cells, and decreased PD-L1 levels in ovarian cancer and breast cancer cells [40–42]. This prompted us to look beyond physiological miR-200c-3p functions and shift our focus to investigating potential artificial effects triggered by the miR-200c-3p mimic A.

The synthetic, double-stranded miRNA mimics consist of the mature miRNA sequence and a complementary passenger strand. Upon entering the cells, the passenger strand is cleaved and the miRNA is incorporated into the RNA-induced silencing complex (RISC). Subsequently, the miRNA guides the RISC to target mRNAs in a sequence-dependent manner, leading to their translational repression. Therefore, by artificially increasing mature miRNA levels through transfection of miRNA mimics, miRNA functions can be studied. Nonetheless, although this technology has been extensively used since its first implementation in 2007, many uncertainties still exist. Doubts are expressed regarding the miRNA mimic design, transfection conditions, passenger strand functions, and most importantly the induction of unintended effects [43,44].

In an effort to dissect underlying mechanisms leading to the non-specific upregulation of ICs, we focused on the cells’ innate immune response. We noticed that transfection of miR-200c-3p mimic A induced the expression of IFN-β and type III IFNs. Moreover, transfected cells displayed an upregulation of genes critically involved in the cellular antiviral response (MX1, OAS1, PKR) and an increase in endosomal (TLR3) as well as cytoplasmic dsRNA sensors (MDA5, RIG-I). Additionally, we observed a substantial inhibition of cellular growth and induction of apoptosis. These effects were not visible in cells transfected with miR-141-3p mimic A and miR-200c-3p mimic B, nor in stable miR-200c-3p overexpression cell lines. Collectively, these results strongly indicate that cells initiated an extensive antiviral programme including interferon response upon recognizing the double-stranded miR-200c-3p mimic A.

In line with our observations, Goldgraben et al. reported effects on interferon response when using miScript miRNA mimics in breast cancer cell lines [45]. When they transfected cells with various 23 nucleotide (nt)-long miRNA mimics, they noticed an induction of dsRNA response and IFNB1 expression, whereas miRNA mimics smaller than 23 nt did not exert these effects. These length-dependent effects might explain why we see an induction of IFNs with the 23 nt-long miScript miR-200c-3p mimic A, but not with the 22 nt-long miScript miR-141-3p mimic A. Furthermore, when Goldgraben et al. mutated the seed sequence of the passenger strand, off-target effects were completely abolished, implying that the non-specific effects were at least partly dependent on the passenger strand. This could be the reason for the contrasting cellular reactions to miScript miR-200c-3p mimic A and mirVana miR-200c-3p mimic B. While the miScript mimics A are strictly unmodified dsRNA molecules, the mirVana mimics B contain chemical modifications to minimize passenger strand effects. These modifications might result in a more effective removal of the passenger strand from the intracellular miRNA mimic pool, thereby circumventing dsRNA sensing and the consequent induction of an antiviral response.

In our study, we highlight another critical component shaping non-specific miRNA mimic effects: the concentration of the mimic. While we observed a strong induction of IFNs and ICs using between 5 and 20 nM miR-200c-3p mimic A, these effects were mostly absent with concentrations below 5 nM. It is conceivable that the artificial supply of exogenous mature miRNAs through transient miRNA mimic transfection overloads the capacities of the cellular silencing machinery above certain concentrations. This means that while the majority of transfected miRNAs is correctly loaded into the RISC, a part of intracellular miRNA mimics cannot be processed. These remaining, double-stranded RNA molecules might then be recognized by dsRNA sensors and initiate the antiviral response. In line with this hypothesis, Thomson et al. reported that the detected level of miRNAs after transient mimic transfection does not reflect the actual functional miRNA levels. Through immunoprecipitation and fluorescent labelling experiments, they realized that only a fraction of transfected miRNAs are incorporated into the RISC, whereas most of the intracellular mimics localized with or adjacent to lysosomes [44]. Since it is known that dsRNA sensors like TLR3 are widely distributed across endosomal and lysosomal compartments, it seems plausible that this facilitates the recognition of unprocessed, double-stranded mimics [46]. Jin et al. highlight a further danger of using high concentrations of miRNA mimics [43]. When they transiently transfected miRNA mimics into HeLa cells, they observed a concentration-dependent accumulation of high molecular weight RNA species and non-specific alterations in gene expression. Interestingly, while they noticed a moderate upregulation of IFN-β, there was no induction of genes associated with antiviral response. This suggests that miRNA mimics can also lead to artificial effects independent of triggering a cells’ antiviral programme. Of note, Jin et al. used miRIDIAN microRNA mimics (Dharmacon®) for their studies. This serves as evidence that non-specific effects are not restricted to miScript miRNA mimics, but might also extend to miRNA mimics from other manufacturers. In addition to miRNA mimics, there are reports of certain siRNA triggering similar unintended effects. Kleinman et al. describe how siRNAs can non-specifically trigger TLR3 signalling, with wide-ranging consequences in various experimental models [47,48]. In a similar fashion, Cho et al. observed that siRNA-TLR3 interaction led to detrimental artificial effects on the circulatory systems [49]. Together, these studies demonstrate that our findings might not only be relevant in the context of miRNA mimics, but may also apply to the transfection of further RNA species like siRNAs.

Our findings have implications for previously published immune-related studies using miRNA mimics, as misinterpretations based on similar non-specific miRNA mimic effects might have occurred in the past. Beyond this, it is important to ensure robust and reproducible results for future transient miRNA overexpression experiments. To this end, we argue that a preliminary screening of expressional changes of IFNs and antiviral response genes like MX1 and OAS1 upon miRNA mimic transfection would be advisable. This initial test would enable an early detection of immune-related, artificial effects. An adequate adaptation of the used miRNA mimic and transfection conditions would consequently prevent misinterpretations of physiological miRNA functions. Furthermore, a validation of transient miRNA overexpression results with stable overexpression cell models seems indispensable.

While our study focusses on miRNA overexpression *in vitro*, this miRNA mimic-dependent induction of immune responses also has potential relevance in the clinical setting. With miRNA-based therapeutic interventions emerging, the focus lies on efficiently and safely transporting miRNA mimics. There are numerous ongoing clinical trials evaluating miRNA therapeutics for cancer treatment [50]. Albeit considerable progress is being made, immune-related side effects are a serious problem. For example, a phase I clinical study of MRX34, a liposomal miR-34a mimic, was prematurely terminated because of severe immune-related side effects causing the death of four patients [51,52]. Although the causative link is unclear, it is conceivable that our observed non-specific effects could play a role. While this is highly speculative, the capability of miRNA mimics inducing the innate immune system should be taken into account when designing miRNA mimic therapeutics. Furthermore, with miRNA-based therapeutic strategies aiming to counteract tumour immune evasion mechanisms, our findings showing a miRNA mimic-dependent upregulation of immune checkpoints should be considered [53].

Conclusively, our data demonstrates the capability of miRNA mimics to drastically mislead interpretations of miRNA functions. While miR-200c-3p has no significant impact on the expression of PD-L1, LGALS9, and IDO1 in BTC, this interpretation was challenged by the non-specific effects of the miR-200c-3p mimic A. This molecule triggers a dsRNA-dependent antiviral interferon response with wide-ranging consequences spanning from an upregulation of immune checkpoints to an inhibition of cellular growth and induction of apoptosis. Without methodical investigation, these effects might be falsely attributed to physiological miR-200c-3p actions, thereby dangerously distorting the interpretation of miRNA gain-of-function experiments. This seems especially important in the context of immune checkpoint regulation. Most importantly, this does not only apply for *in vitro* miRNA studies, but also needs to be considered for the design of miRNA mimic therapeutics.

## Supplementary Material

Supplemental Material

## Data Availability

The data that support the findings of this study are available from the corresponding author, MP, upon reasonable request.
