# Supplementary material for: MicroRNA mimics can distort physiological microRNA effects on immune checkpoints by triggering an antiviral interferon response
Source: RNA Biol. 2022 Dec 5;19(1):1305–15. doi: 10.1080/15476286.2022.2152978 (PMC9728468; doi:10.1080/15476286.2022.2152978)
Supplement: Supplemental Material [file KRNB_A_2152978_SM5385.docx]

Supplementary Material

# Supplementary Tables

Supplementary Table 1. Primer sequences used for RT-qPCR analyses.

| Target | Forward primer (5’ 🡪 3’) | Reverse primer (5’ 🡪 3’) |
| --- | --- | --- |
| Keratin 8 | ATGTTGTCCATGTTGCTTCG | CCAGGAGAAGGAGCAGATCA |
| E-cadherin | TGAAGGTGACAGAGCCTCTGGAT | GGGTGAATTCGGGCTTGTT |
| N-cadherin | GACGGTTCGCCATCCAGAC | TCGATTGGTTTGACCACGG |
| COL3A1 | ATATTTGGCATGGTTCTGGC | TGGCTACTTCTCGCTCTGCT |
| Vimentin | CCTTGAACGCAAAGTGGAATC | GACATGCTGTTCCTGAATCTGAG |
| ZEB1 | GATGATGAATGCGAGTCAGATGC | ACAGCAGTGTCTTGTTGTTGT |
| ZEB2 | CAAGAGGCGCAAACAAGCC | GGTTGGCAATACCGTCATCC |
| IDO1 | GCCAGCTTCGAGAAAGAGTTG | ATCCCAGAACTAGACGTGCAA |
| LGALS9 | GGACGGACTTCAGATCACTGT | CCATCTTCAAACCGAGGGTTG |
| PD-L1 | TGGCATTTGCTGAACGCATTT | TGCAGCCAGGTCTAATTGTTTT |
| PD-L2 | ATTGCAGCTTCACCAGATAGC | AAAGTTGCATTCCAGGGTCAC |
| CD80 | AAACTCGCATCTACTGGCAAA | GGTTCTTGTACTCGGGCCATA |
| CD86 | CTGCTCATCTATACACGGTTACC | GGAAACGTCGTACAGTTCTGTG |
| B7H2 | GCAGCCTTCGAGCTGATACTC | GTTTTCGACTCACTGGTTTGC |
| B7H3 | CACTGTGGTTCTGCCTCACA | AGATGAGGTTGAGCTGTGCC |
| TNFRSF14 | GTGCAGTCCAGGTTATCGTGT | CACTTGCTTAGGCCATTGAGG |
| HLA-A | CATCTCTGACCATGAGGCCA | GGCAGGTGTATCTCTGCTCC |
| TNFSF9 | GGCGTCCATCTTCACACTGA | CACCCAGGCTGGACGTTATT |
| TNFSF4 | CCAGGCCAAGATTCGAGAGG | CCGATGTGATACCTGAAGAGCA |
| TNFRSF5 | ACTGAAACGGAATGCCTTCCT | CCTCACTCGTACAGTGCCA |
| LGALS9 | GGACGGACTTCAGATCACTGT | CCATCTTCAAACCGAGGGTTG |
| B7H5 | ACGCCGTATTCCCTGTATGTC | TTGTAGAAGGTCACATCGTGC |
| B7H4 | TCTGGGCATCCCAAGTTGAC | TCCGCCTTTTGATCTCCGATT |
| CD47 | TCCGGTGGTATGGATGAGAAA | ACCAAGGCCAGTAGCATTCTT |
| LGALS3 | ATGGCAGACAATTTTTCGCTCC | GCCTGTCCAGGATAAGCCC |
| HLA-DPA1 | ATGCGCCCTGAAGACAGAATG | ACACATGGTCCGCCTTGATG |
| CLEC4G | AGTCCTTTGGGCTGTGATTCT | AGGCGTTTGTCCTCAGCAG |
| HMGB1 | TTTGTGCAAACTTGTCGGGAG | TTCCACCTCTCTGAGCACTT |
| CEACAM1 | CCACTTCACAGAGTGCGTGT | CCAAAAAGTTGCTGGGGCAG |
| IFNB1 | CAACCTTTCGAAGCCTTTGC | TCCCATTCAATTGCCACAGG |
| IFNL1 | TCTGAGAACGTCAACCCACC | TATGTCTCAGTCAGGGCTGC |
| IFNL2 | GAATTGTGTTGCCAGTGGGG | CATTTTCCTGGAGGTGAGTTGG |
| IFNL3 | TGAAACTAGACATGACCGGGG | GAGACAGGGACTTGAACTGGG |
| IFNAR1 | GCACACACCATGGATGAAAAGC | GCCAAATTTTAGAGGTATTTCCTGG |
| IFNAR2 | ATAGCAAAGATGCTTTTGAGCC | TGCAAGATTCATCTGTGTAATCAGG |
| IFNLr1 | TGGCCTATCAGAGCTCTCCC | TCAGGATCTCCTCCGTCTGG |
| IL10RB | ACAACCCATGACGAAACGG | GAATTCCTAGGGGAGAAGGCG |
| RIG-I | GACCCTACCTACATCCTGAGC | CTTCATAAAGTCCAGAATAACCTGC |
| MDA5 | TTGGCAGAAGGAAGTGTCAGC | TTCTTCCCTTCCAAGGCTGG |
| TLR3 | AAAACCTTTGCCTTCTGCACG | TTCCAGCTGAACCTGAGTTCC |
| OAS1 | AAGCTCAAGAGCCTCATCCG | TCCAAGACCGTCCGAAATCC |
| MX1 | GGACATCGCAAAAGCTGATCC | GTTGTTCTCAGCCACCGAGC |
| PKR | CTTCCATCTGACTCAGGTTTGC | TCTTCTTCCCGTATCCTGGTTGG |
| GAPDH | AAGGTCGGAGTCAACGGATTT | ACCAGAGTTAAAAGCAGCCCTG |
| U6 | CTCGCTTCGGCAGCACA | AACGCTTCACGAATTTGCGT |

Supplementary Table 2. Primary and secondary antibodies used for immunoblot analyses.

| PRIMARY ANTIBODIES | | | | | | |
| --- | --- | --- | --- | --- | --- | --- |
| Target | **Antibody** | **Dilution** | **Species** | **Identification** | **RRID** | **Company** |
| E-cadherin | E-cadherin (G-10) | 1:1,000 | Mouse | sc-8426 | AB_626780 | Santa Cruz Biotechnology, Inc. |
| ZEB1 | TCF8/ZEB1 (D80D3) Rabbit mAb | 1:1,000 | Rabbit | #3396 | AB_1904164 | Cell Signaling Technology® |
| IDO1 | IDO Recombinant Rabbit Monoclonal Antibody (7H8L17) | 1:2,000 | Rabbit | #702743 | AB_2716890 | Thermo Fisher Scientific |
| PD-L1 | PD-L1/CD274 Monoclonal antibody | 1:5,000 | **Mouse** | **66248-1-Ig** | AB_2756526 | Proteintech Group, Inc. |
| Cofilin | Anti-Cofilin antibody | 1:5,000 | **Rabbit** | ab42824 | AB_879739 | Abcam |
| SECONDARY ANTIBODIES | | | | | | |
| Antibody | | **Dilution** | **Species** | **Identification** | **RRID** | **Company** |
| Rabbit Anti-Mouse IgG (Light Chain Specific) (D3V2A) mAb (HRP Conjugate) | | 1:2,000 | **Rabbit** | #58802 | AB_2799549 | Cell Signaling Technology® |
| Peroxidase AffiniPure Goat Anti-Rabbit IgG antibody | | 1:10,000 | **Goat** | **111-035-144** | AB_2307391 | Jackson ImmunoResearch |

# Supplementary Figures


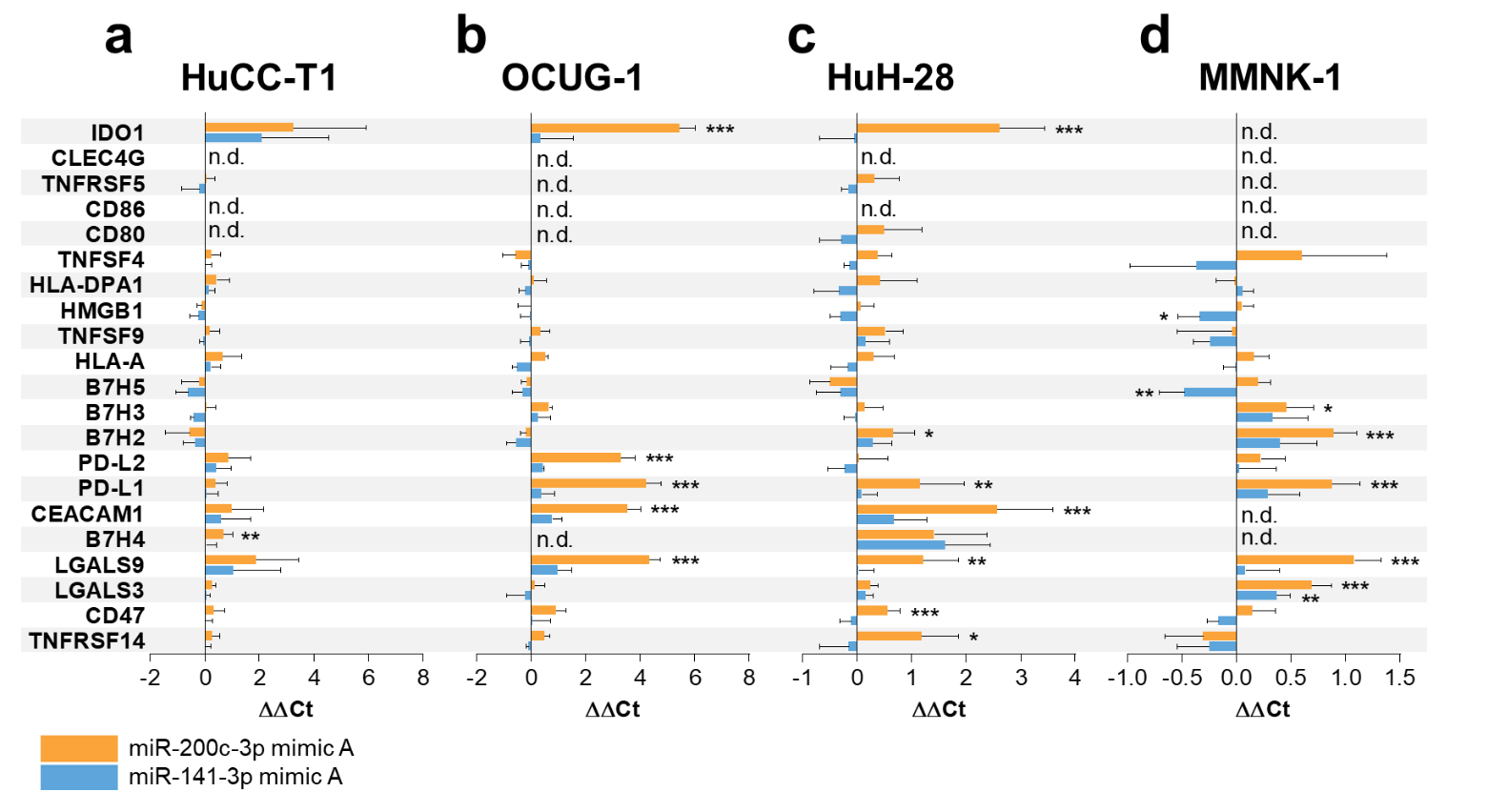


**Supplementary Figure 1.** **Influence of miR-200c-3p mimic A and miR-141-3p mimic A transfection on the expression of immune checkpoints in BTC cells. (A)** HuCC-T1 (n=5), **(B)** OCUG-1 (n=3), **(C)** HuH-28 (n=5), and **(D)** MMNK-1 (n=5) cells were transiently transfected with 10 nM miScript miR-200c-3p mimic A (yellow bars) or miScript miR-141-3p mimic A (blue bars) and AllStars Neg. Ctrl. for 48 hours. Expression levels of various immune checkpoints were analyzed via RT-qPCR. The mean of GAPDH+U6 was used for the normalization of mRNA levels. Differences in expression were evaluated using the ΔΔCt method. Data is presented as mean + SD. Statistical analysis was performed using one-way ANOVA with Tukey post-test. *p<0.05, **p<0.01, ***p<0.001. n.d.=not detectable (Ct≥35).


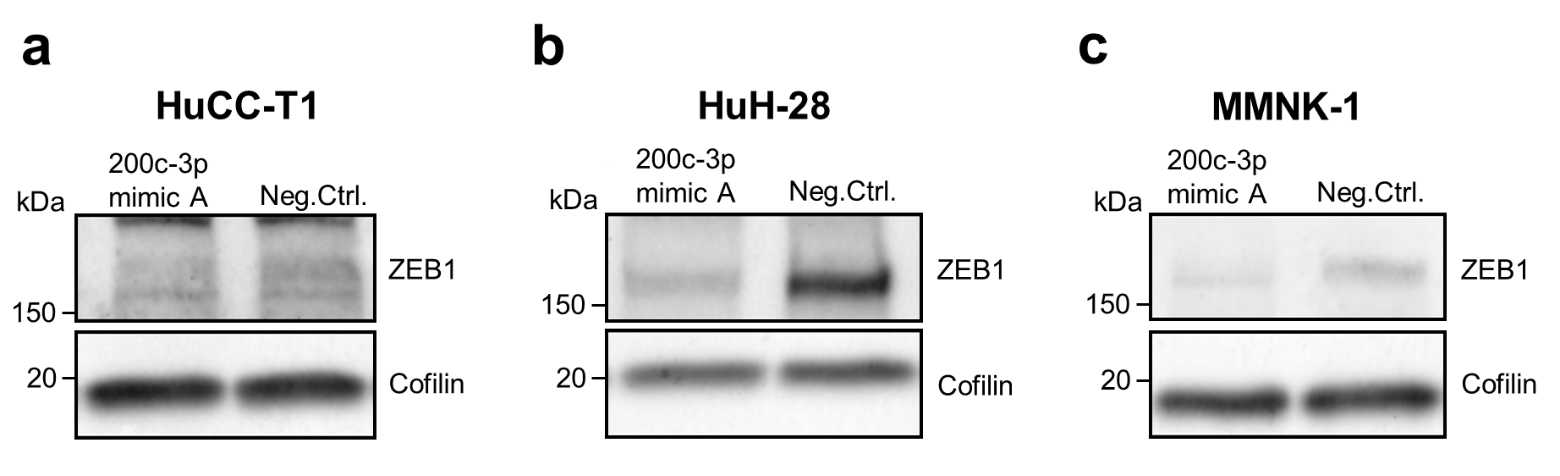


**Supplementary Figure 2.** **Transient overexpression of miR-200c-3p leads to a downregulation of ZEB1. (A)** HuCC-T1 and **(C)** MMNK-1 cells were transfected with miScript miR-200c-3p mimic A for 72 hours, **(B)** HuH-28 for 48 hours, and resulting changes in ZEB1 protein were analyzed via Western Blot where applicable. Cofilin was used as loading control.


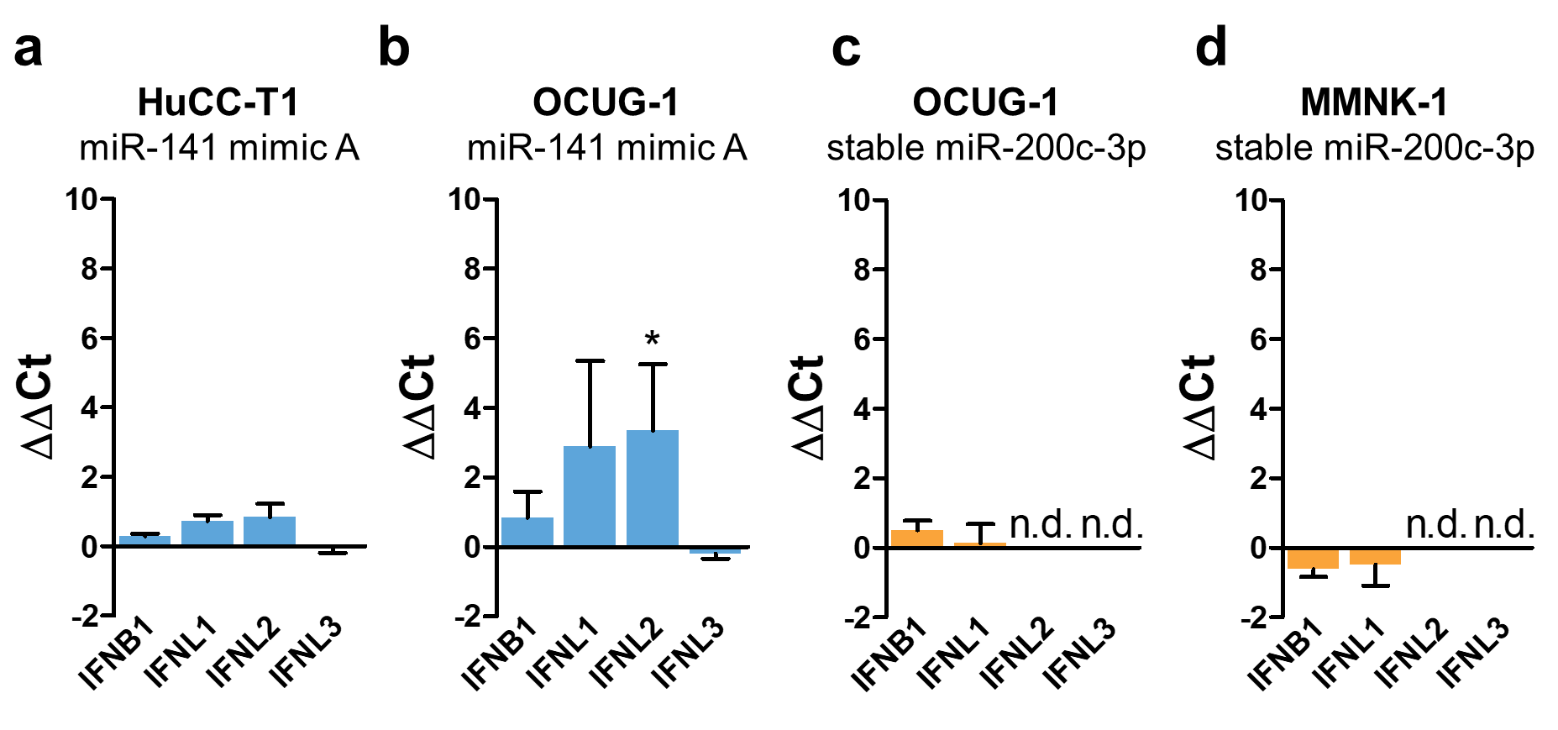


**Supplementary Figure 3.** **There is no upregulation of interferons by miR-141-3p mimic A transfection, nor in stable miR-200c-3p overexpression cell lines. (A, B)** HuCC-T1 (n=3) and OCUG-1 (n=3) cells were transiently transfected with 10 nM miScript miR-141-3p mimic A and corresponding negative control for 48 hours. Expression levels of IFNB1, IFNL1, IFNL2, and IFNL3 of transfected cells and of **(C, D)** stable miR-200c-3p overexpression OCUG-1 (n=3) and MMNK-1 (n=3) cells were analyzed via RT-qPCR. The mean of GAPDH+U6 was used for the normalization of mRNA levels. Differences in expression were evaluated using the ΔΔCt method. Data is presented as mean + SD. Statistical analysis was performed using unpaired two-tailed t-test. *p<0.05. n.d.=not detectable (Ct≥35).


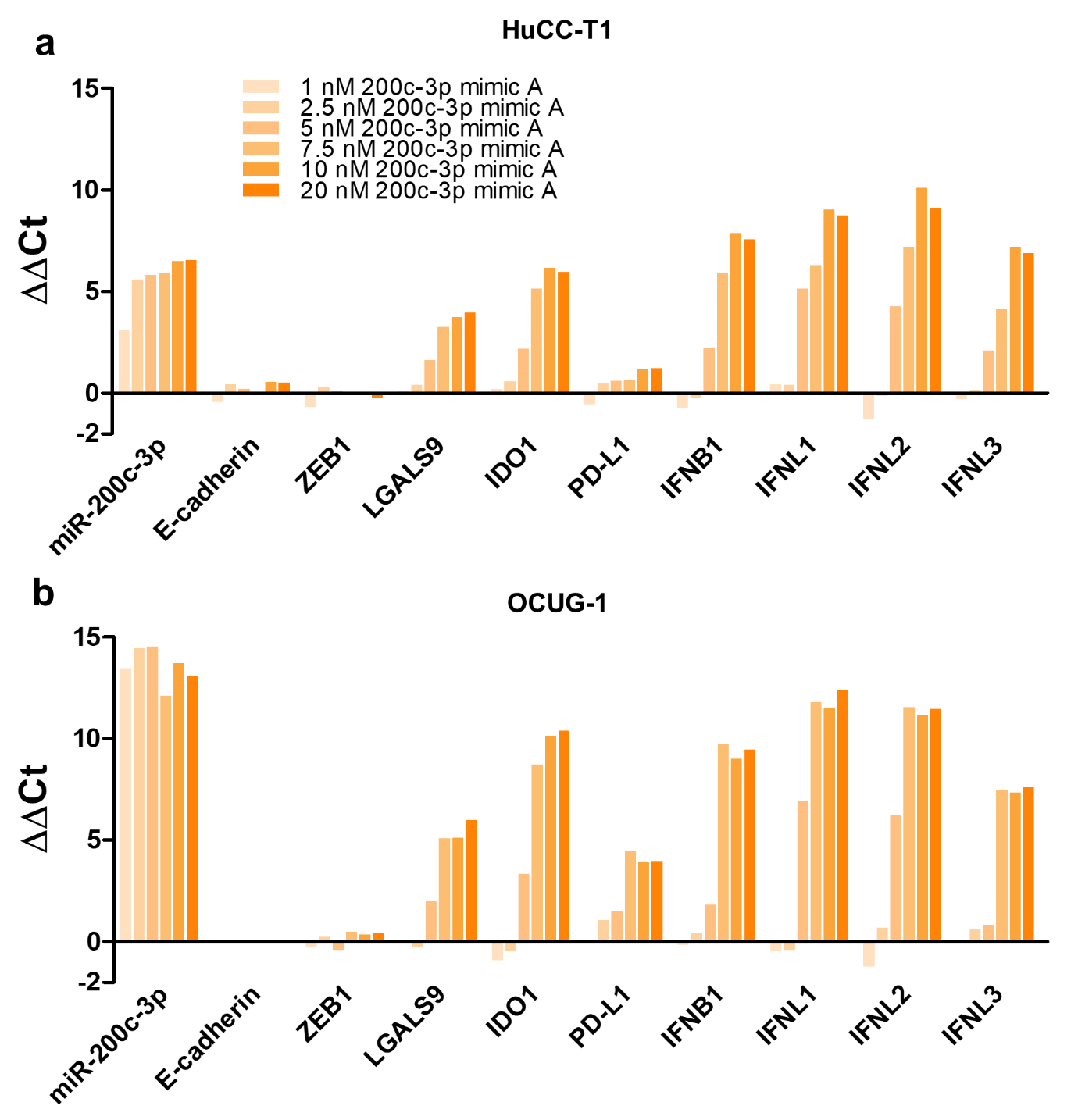


**Supplementary Figure 4.** **miR-200c-3p mimic A transfection leads to a concentration-dependent upregulation of immune checkpoints and interferons. (A)** HuCC-T1 (n=1) and **(B)** OCUG-1 (n=1) cells were transiently transfected with 1-20 nM miScript miR-200c-3p mimic A or AllStars Neg. Ctrl. for 48 hours and expression levels of miR-200c-3p, E-cadherin, ZEB1, LGALS9, PD-L1, IDO1, IFNB1, IFNL1, IFNL2, and IFNL3 were analyzed via RT-qPCR. RNU6B was used for normalization of miRNA levels, the mean of GAPDH+U6 for the normalization of mRNA levels.
